# Implementing Replication of Objects in DOORS—The Object-Oriented Runtime System for Edge Computing

**DOI:** 10.3390/s21237883

**Published:** 2021-11-26

**Authors:** Dorin Palanciuc, Florin Pop

**Affiliations:** 1 Computer Science and Engineering Department, Faculty of Automatic Control and Computers, University Politehnica of Bucharest, 313 Splaiul Independenței, 060042 Bucharest, Romania; dorin.palanciuc@stud.acs.upb.ro; 2 National Institute for Research & Development in Informatics—ICI, 8-10 Bulevardul Mareșal Alexandru Averescu, 011555 Bucharest, Romania

**Keywords:** replication, partitioning, object-oriented, message passing, edge

## Abstract

Aiming for simplicity and efficiency in the domain of edge computing, DOORS is a distributed system expected to scale up to hundreds of nodes, which encapsulates application state and behavior into objects and gives them the ability to exchange asynchronous messages. DOORS offers semi-synchronous replication and the ability to explicitly move objects from one node to another, as methods to achieve scalability and resilience. The present paper gives an outline of the system structure, describes how DOORS implements object replication, and describes a basic set of measurements, yielding an initial set of conclusions for the improvements of the design.

## 1. Introduction

The Distributed Object Oriented Runtime System (DOORS) proposes a minimal set of services needed for a solution implementer to address a geographically distributed problem. Obvious examples of problems targeted by DOORS are telemetry, SCADA, IoT, maintenance, and monitoring of mission-critical systems.

The set of DOORS services is based on the fundamental OOP concepts of class and object. The former is encapsulating data structures (system state) and the code acting upon them (system behavior), while the latter is an individual instance of a class. DOORS objects are also able to exchange asynchronous messages (with other objects or with external clients), and the system ensures message transit and delivery. When communication faults occur, the system handles queuing and re-sending of not-yet-confirmed messages. On top of the fundamentals, the system offers: an event model, storage of objects, and replication and partitioning of objects across the available set of nodes, as well as the ability that multiple versions of the same class to coexist and run side by side. Last but not least, DOORS provides services for explicit relocation of objects from one node to another, which allows rational use of the available resources, flexibility in operation, and the ability to move processing capacity closer to the source of data, minimizing latency and rendering DOORS a viable solution for edge computing [1,2].

The reasons for which DOORS employs object orientation, as well as its main features, are described in Reference [3]. In “traditional OOP fashion”, DOORS objects encapsulate state and behavior, and our major source of inspiration is the Smalltalk language, as emphasized in Reference [4], where we also detail how DOORS achieves consistency and availability. A detailed analysis of object migration in DOORS and its impact on scheduling for execution can be found in Reference [5]. The present paper presents the replication of objects in DOORS, a mechanism that offers high availability of the proposed systems.

Considering an operating system for the reference architecture (e.g., Linux), the default scheduling behavior of the kernel is out of the control of the system implementer for our proposed solution. Our approach may employ means for explicitly allocating tasks to CPU cores using the concept of abstract object oriented runtime system for heterogeneous parallel architecture [6]. This explicit allocation is achievable by setting the affinity of individual threads.

The paper is structured as follows. Section 2 presents the main target of DOORS approach. Then, Section 3 analyzes the orientation towards edge computing. Section 4 describes in details the proposed solution, and Section 5 highlights the experimental results. The paper ends with con conclusions and future work.

## 2. The Target of DOORS

As stated in Reference [4], the design of DOORS is focused on a subclass of distributed problems for which edge-computing solutions are viable alternatives.

**Figure 1:**
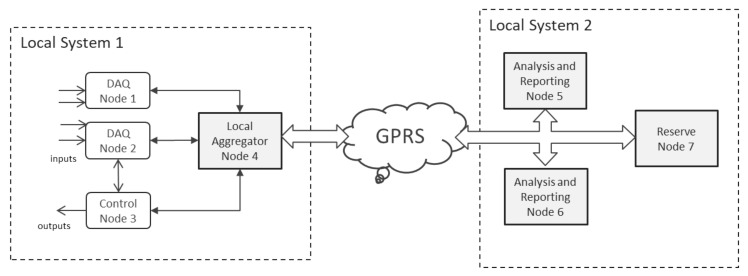
Example of geographically distributed DOORS installation.

Figure 1 depicts a typical geographical distributed system, with remote nodes, specialized in data acquisition or control, as well as central nodes, specialized in data aggregation and analytics. The system contains local control loops, as well as a centrally located cluster of computing capacity. Last but not least, the system features unreliable network connections toward remote nodes.

Basing our analysis on [7], although Big Data is not in the focus of DOORS, the premise for parallelism created by the ability to partition objects across the set of available nodes qualifies our proposal for addressing the heterogeneity of data-sets, processes, or even system infrastructure [8], which is often encountered in “traditional” analytics implementations.

## 3. Orientation towards Edge Computing

Edge Computing, as well as the partially overlapping concept of Fog Computing, share many of the design goals of DOORS, due to their orientation towards decentralization and migration of control towards “system periphery” [9,10,11,12]. Reference [2] introduces the subject, noting the recurrent and pervasive struggle between the forces of centralization and the forces of decentralization, and advocating for a new, post-cloud wave of decentralization. The generally accepted definition of fog computing, “a paradigm that extends Cloud computing and services to the edge of the network”, is targeting real-life scenarios in the area of Assisted Living, as exemplified by Reference [13].

Based on the planned features, we expect that our proposed system architecture fits in this area and allows for, hopefully, simple implementations of low-latency, decentralized event-oriented solutions. The design shall provide solutions for concurrency control, resilience through redundancy, and maintaining an adequate trade-off between consistency and availability, during system segmentation events.

We acknowledge the approach of Drop Computing, which is oriented towards ad-hoc collaboration and aims for higher levels of scalability [14,15,16]. However, we postulate that mission-critical systems are not based on ad-hoc configurations, node turnover is extremely limited if tolerated at all, and, therefore, we concentrate the research effort on a simplified architecture, as described in the following sections [1,17].

## 4. Proposed Solution

Following subsections describe the solutions adopted by DOORS for the realization of its key distributive features: replication and partitioning. The former refers to the act of maintaining multiple copies of the same object in distinct location (on different nodes in our case), while the latter denotes the “spreading out” the objects over the available nodes, such that each of them is hosted on a single node. The partitioning of objects in DOORS has the mathematical meaning of the partitioning operation applied to the set of objects hosted by the system, in the sense that each node hosts one of the resulted subsets.

### 4.1. The Structure of DOORS Objects

**Figure 2:**
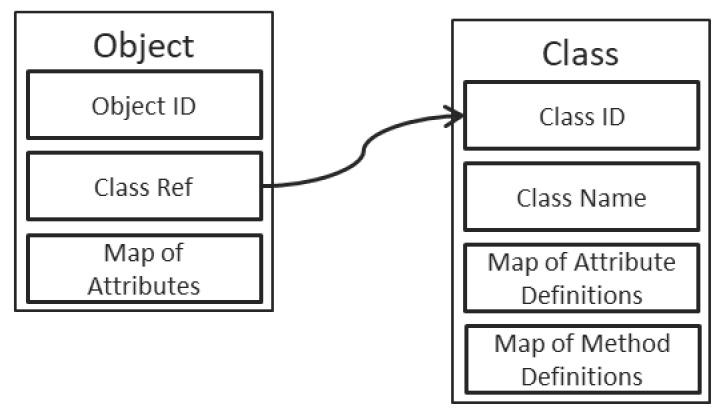
Structures of objects and classes in DOORS.

**Figure 3:**
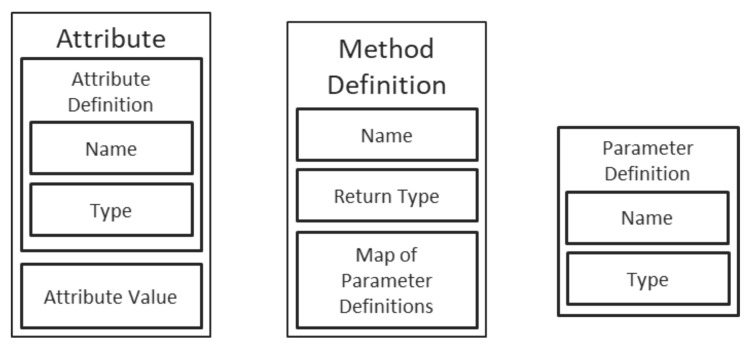
Definitions for attributes, methods and parameters.

As depicted in Figure 2, the objects have a straightforward structure, containing first and foremost an unique ID (which is a GUID stored as a string of characters in the reference implementation), followed by a reference to the definition of the corresponding class and completed by a map of the object’s attributes. The keys in the map are the names of the attributes, while the values in the map are dedicated structures, as depicted in Figure 3. Each attribute contains, therefore, a description, composed of a name, a type, and a value.

All the maps utilize a straightforward implementation of a hash table (not considered relevant for the scope of the present material), while the values are void pointers, referring to the actual data fields, stored on the heap.

### 4.2. Types of DOORS Messages

#### 4.2.1. Notifications

The simplest form of inter-node communication is one in which we choose to have explicit and immediate confirmation of each message. We analyze the situation in which the connection between the two nodes is lost between the moment the notification is being sent and the moment when the receipt confirmation is received. As it shall be seen in the analysis below, depending on the exact moment of fault occurrence, we might have no notification exchanged, exactly one notification exchanged, or multiple identical notifications exchanged. Depending on the significance associated to the receipt of the respective notification, the exchange of multiple messages referring to the same real-life event may be undesirable [18].

#### 4.2.2. Loss of Communication during Notification Transfer

**Figure 4:**
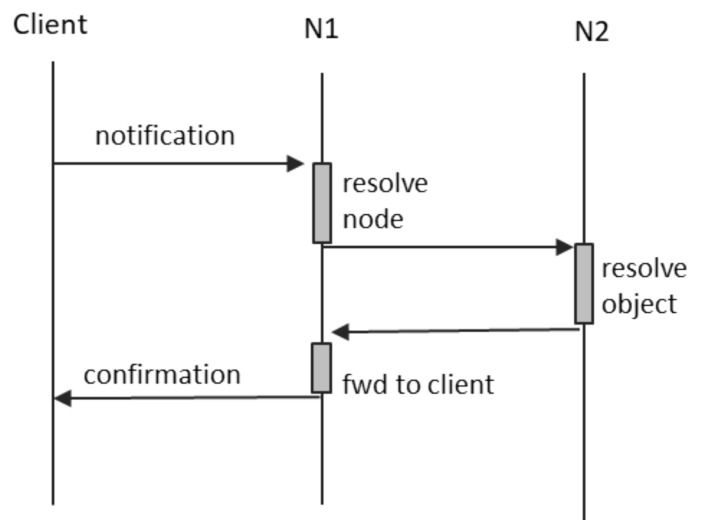
Notifications.

Figure 4 depicts the communication scenario occurring in the case of notification messages. N1 is the node to which the client is directly connected, and N2 is the node hosting the object targeted by the notification.

The most relevant aspects are the following:

- N1 uses its local resources (presented below) in order to resolve the node on which the target object resides;
- N2 uses its local resources in order to resolve the target objects;
- N1 caches the ID of the client sender, so that it can determine where to forward the receipt confirmation when it is available; and
- both N1 and N2 use a message digest in order to uniquely identify notifications and associate them with the ID of their sender.

**Figure 5:**
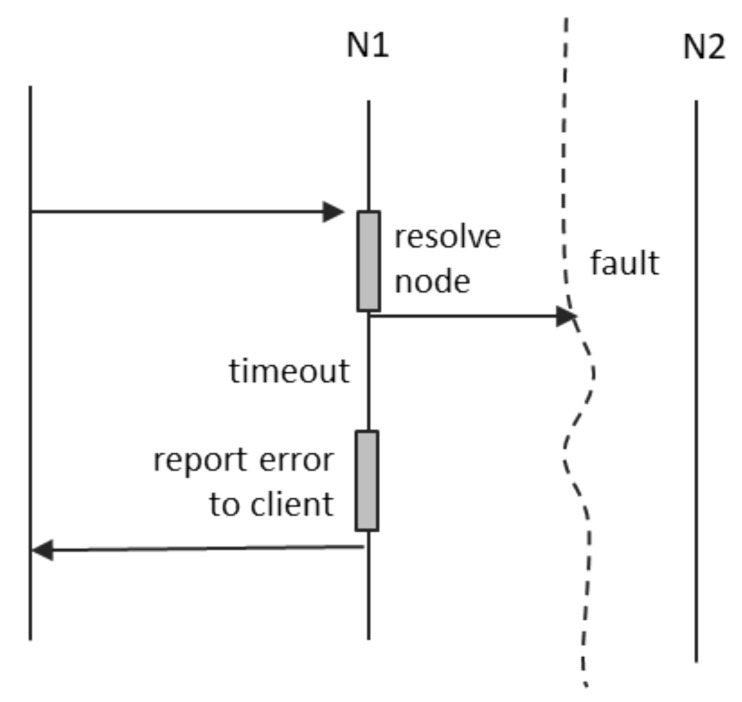
Notification fault.

A connection fault between the client and N1 means that the notification will not reach N1, and no confirmation is ever received. A fault on the path between N1 and N2 means that the notification will not reach its destination, and, again, no confirmation is available. In this case, we have two possible alternatives:

- N1 may store the notification locally, as an “offline message”, and place it in the send queue for when the connection to N2 is restored. A conservation period may be defined, after which N1 shall discard the cached notification.
- N1 may detect the loss of connection to N2 and send an error message back to the client. This case is depicted in Figure 5.

If a fault occurs between N1 and N2, node N1 will not be able to ascertain whether the notification was indeed received by the destination object. This is the reason why the inter-node communication protocol implements a mechanism for idem-potency: even when any of the nodes receives the same message twice, it must be able to ascertain in a quick and efficient manner that the two messages refer to the same event. The solution that we propose is based on the fact both nodes may compute a quasi-unique ID of any message—a simple digest. This ID is used by the actors involved as follows:

- N1 computes IDs for all messages that it needs to forward to other peers and stores locally the IDs of all messages not yet confirmed, each of them in pair with the ID of the notification sender. Whenever it receives a notification confirmation from a peer, it shall compute its ID and then look into the list of locally stored IDs. If already present, it shall forward the confirmation to the corresponding sender. If the computed ID is not in the locally stored list, N1 shall discard the confirmation without any forward.
- N2 computes the IDs of all incoming notifications and includes them in their respective confirmations.
- The client shall also maintain a list of the IDs of all notifications sent and not yet confirmed. When a confirmation arrives, it shall compare the ID received with the one stored locally and, therefore, be able to track which of its notifications have been successfully received.

We shall observe that, in the case of all confirmed notifications, the sender can be certain of their reception, while, in the case of not confirmed ones, it may be that at least some of them reached their intended destination. While this aspect may not be important for notifications, it becomes relevant in the case of service requests, as described in the next section.

#### 4.2.3. Service Requests

This type of inter-node communication is the most usual (and useful) form of interaction in a distributed system: node A is sending a message to node B and expects an answer in return. DOORS supports asynchronous behavior, so the response of node B may arrive at a much later moment in time. Besides the already discussed and addressed challenge of “at-least-once” transmission, we have to address additional problems:

- pairing up a request with a much later answer (potentially received after several other requests, maybe even “out-of-order”);
- deciding when to “stop waiting” for an answer;
- determining whether a request produced any effects on the destination object; and
- de-duplicating multiple responses.

Again, the unique identification of the request (by the receiving node) and the inclusion of the ID into the response shall be used for de-duplication and pairing requests with responses. However, for the timing related challenges, we rely on the conclusion of Fischer and Linch in Reference [19] that, on fully asynchronous systems, consensus is impossible to obtain in the presence of node crashes. We, therefore, time-box the period in which an object must reply. If this time period expires, both the sender and the receiver must assume that the service request fails and act accordingly (the sender may choose to abandon or re-attempt the request, and the receiver may choose to cancel the service execution and roll back any partial changes that may have been performed).

### 4.3. Loss of Communication during Service Requests

**Figure 6:**
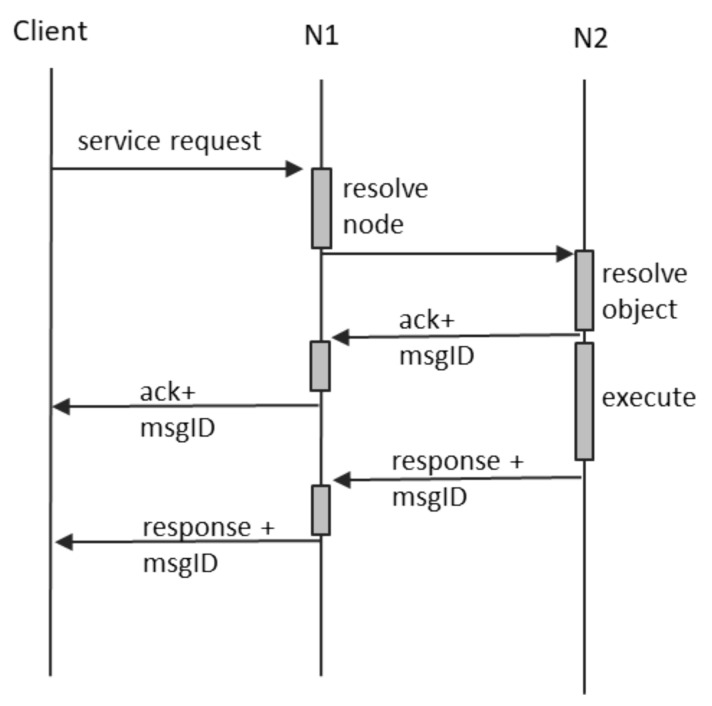
Service request scenario.

In DOORS, we assimilate service calls to objects invoking methods of other objects. Object A is sending a message to Object B, which resides on another node in order to trigger some internal computation. So far, it is similar to the notification, save for a very important difference: an answer is expected. This means that, besides the normal receipt confirmation message, Object B shall send back a second response, containing the expected result. The same ID constructed by N1 shall be used in similar manner, to pair up the initial request and the receipt confirmation, as well as with the final response, containing the expected result, as well as to eliminate the potential duplicates at each reception. The normal communication process is depicted in Figure 6.

The asynchronous semantics for service calls in DOORS mean that:

- any client requesting a service from a DOORS object shall receive two answers: an immediate receipt confirmation and a later service response;
- if no receipt confirmation is received in the normal message exchange time window, the client shall assume that the request produced no change of state;
- if the receipt confirmation is received but contains the “DESTINATION OFF-LINE” flag, the client shall assume that the system will re-attempt transmission and execution of service request when the destination returns online, provided that the service window has not expired;
- if the receipt confirmation is received without the “DESTINATION OFF-LINE” flag set, the client shall assume that the system successfully routed the request to the intended object and that the service is executing or shall be executed in the future, during the service window period;
- if the receipt confirmation is received without the “DESTINATION OFF-LINE” flag set, but now response is received during the service window period, the client shall assume that the service execution failed and that now side effects occurred; and
- if the receipt confirmation is received and the final answer is also received, the client shall assume that the service executed and that any potential side-effects are durable.

**Figure 7:**
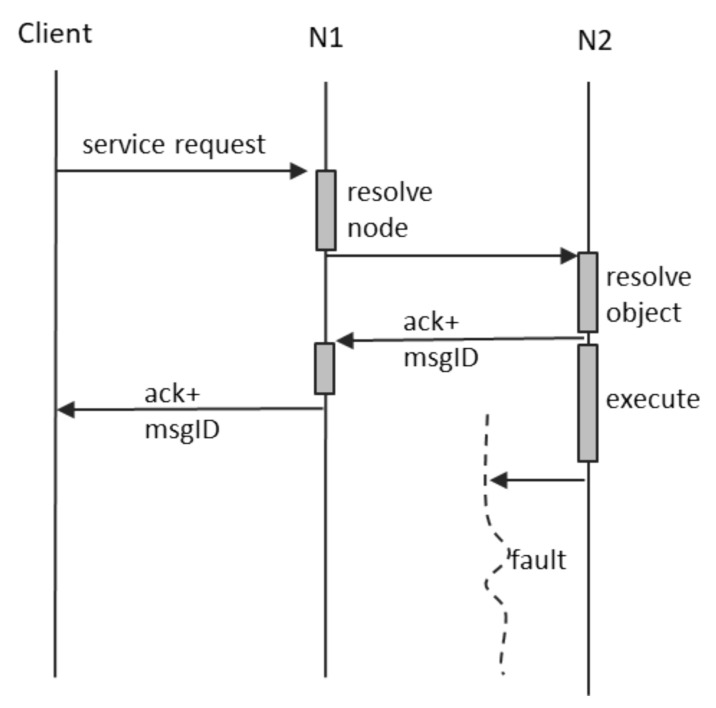
Fault during service request.

If a fault occurs between the client and N1, the system behavior is similar with the case of notifications. In addition, if a fault occurs between N1 and N2 before the receipt confirmation is returned by N2, the system behavior is again similar with the case of notification. The interesting failure scenario is when the fault between N1 and N2 occurs after receipt confirmation, but before the transmission of the final service response. This case is depicted in Figure 7, and it shows that the client ends up not receiving the final service response. The intermediate node N1 performs no further interpretation of this situation and does not create any supplementary messages.

### 4.4. The DOORS Approach on Object Partitioning

Class definitions are replicated on each node, while individual objects are atomically stored—all their attributes are hosted on a single node. Of course, objects may contain as attributes references to other objects, and those referred objects may be hosted on other nodes. The system uses digest values for object IDs; therefore, there is no relationship between any ID value and the node currently hosting an object. Instead, the “current address” of any object is maintained in the Object Dictionary.

#### 4.4.1. Routing of Messages

Routing messages in distributed systems is making sure that they reach their intended destination, taking into account the context of partitioning. There is a continuous spectrum of possible solutions, out of which we mention:

- require that the client entities are aware of the location of each destination object; this would mean that the client must know to which node to connect in order to send the message;
- maintain a dedicated entity, which is in charge with the routing, by maintaining an “address book”; the client must always connect to this dedicated entity; and
- allow the client to connect to any node and implement the “address book” on every node.

We have chosen the latter solution for DOORS, and the Object Dictionary is the globally-replicated “address book”.

#### 4.4.2. Implicit Partitioning and Explicit Balancing

The partitioning policy applied by DOORS is to instantiate any object on the node on which this operation is required. We name this policy “implicit partitioning”. This is the simplest policy to implement, it minimizes the cost of the class instantiating operation, and it also assumes locality, in the sense that objects creating other objects are the most probable clients of those objects; keeping them on the same node decreases the cost of their future exchanges of messages. On the other hand, due to particular conditions, e.g., when all objects are created by a single client, while the client is connected to the same node, we end up having all user-created objects hosted on a single node, creating a “hot spot”, and effectively no partitioning of the set of objects. It may be that the specific needs of a certain concrete implementation dictate other policies, so DOORS also provides system services allowing for explicit migration of any object from one node to another. We shall refer to this as “explicit balancing”, as it is always explicitly performed by the application and not provided generically by the system runtime [20,21].

When objects are being moved as an effect of explicit balancing, the Object Dictionary must be updated and kept in sync on all nodes. This operation must conclude with the consensus of all nodes, such that any message, originating from any of the nodes, is properly routed to its destination, and, due to this, the ability to move objects remains available as long as there is a majority of nodes interconnected.

### 4.5. The DOORS Approach on Object Replication

We define the replication factor as the number of copies of an object which exist at any moment in time on a DOORS instance. These copies have all the same ID and the same values for all attributes. A replication factor of 1 means that each object has a single copy in the system, which means effectively no replication. A greater replication factor means that the system provides redundancy. DOORS implements single-leader replication, meaning that, at any replication factor value, a single copy is “active”, receiving, and processing reads and writes. We consider this copy the primary replica or “master”. All the other copies are named secondary replicas, or “slaves”, and they are only following the evolution of the object state, as occurring on the master. One of the “secondaries” shall take over as primary replica, in case the node hosting the initial master copy becomes unavailable to the majority of peers.

**Figure 8:**
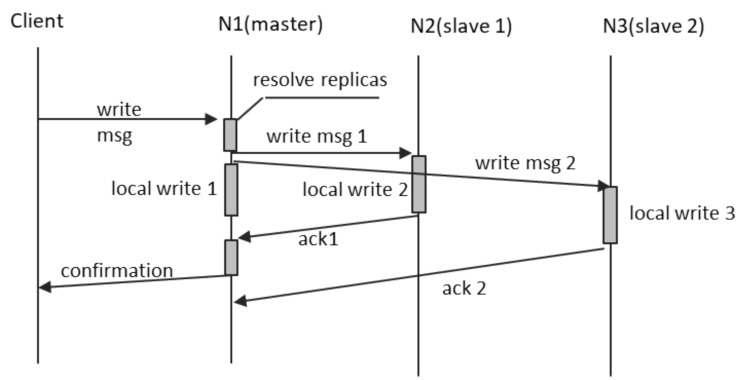
Semi-synchronous replication.

The system implements a variant of single-leader replication, the so-called “semi-synchronous”, depicted in Figure 8, in which only the first replication operation is synchronous, regardless of the replication factor. All reads and writes occur on the primary replica. All write operations are confirmed back to their issuer only after it was propagated on the first secondary replica. For DOORS implementation where the replication factor is greater than 2, all the other secondary replicas shall be updated asynchronously, after the confirmation of the operation.

Confirmation of a write is performed in two ways, based on the way the write is performed:

- for writes requested by the object itself, the write operation shall only return after the confirmation; and
- for writes requested by other objects, which are in fact service request messages, the response message (the second reply message, labeled as “response + msgID” in Figure 6) shall be sent as confirmation.

In the following subsections, we describe the behavior of the system in various relevant situations regarding the replication mechanism, and, in preparation of this, we describe the data structure used for tracking objects within the system: the Object Dictionary.

### 4.6. Tracking Object Replicas

**Figure 9:**
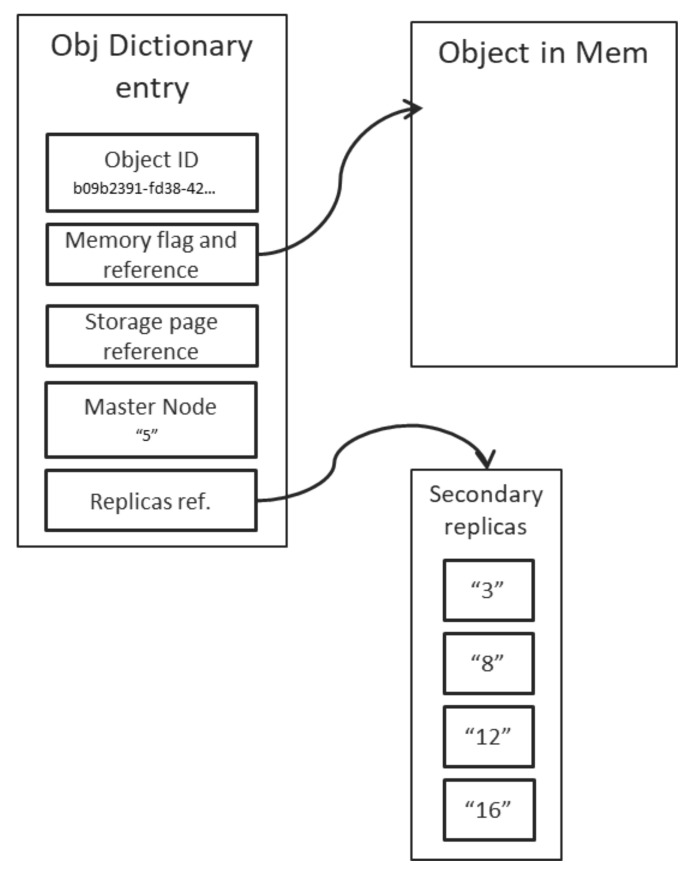
Structure of an Object Dictionary entry.

In addition to tracking the location of the master replica for each object in the system, the Object Dictionary is also maintaining other important data, critical for the correct and efficient management of objects. The structure is outlined in Figure 9, which depicts the following important fields:

- the object ID: in GUID form, it is the key of the dictionary; the implementation shall ensure quick access, by maintaining a sorted key-set;
- memory flag and reference: a Boolean flag, set to *true* if the object is currently in memory; the reference is a local pointer, indicating the location in the main memory of the node where the object can be found;
- storage page reference: the id of the storage page if the object is not in memory, but “swapped-out” to storage instead;
- master node: the id of the node on which the master replica of the object resides; the memory flag and reference shall be populated only for the entries pertaining to objects hosted on the current node; and
- replicas reference: a reference to a sorted list of node ids, indicating which nodes contain the secondary replicas of the object.

### 4.7. Object Creation

Everything begins with a node receiving a service request, for instantiating a class. This request may arrive from an external, directly-connected client, or from another object currently hosted on the current node (as stated above, any DOORS object shall be created on the node on which the request is issued). We consider this to be the primary node, and the major steps performed on it are the following:

1. creation of the master replica of the object: At the end of this step, the structure depicted in Figure 2 is present in the node’s memory, and the GUID of the object is generated.
2. creation of the corresponding Object Dictionary Entry: This step encompasses the random selection of the nodes which shall host the secondary replicas; at the end of it, the structure depicted in Figure 9 shall be present in the Dictionary replica present on the primary node; all the secondary replica entries shall be marked as “invalid”, as the object is not yet replicated.
3. replication of the Object Dictionary Entry: This is done by the primary node by sending dedicated messages, containing the “draft” dictionary entry. The majority of the peers must confirm this before the operation proceeds.
4. creation of the first secondary replica: This is done by the master node by sending a dedicated message to the node which shall contain the secondary replica, mostly similar to the initial instantiating message, except that it contains the already generated GUID of the object. The secondary node must confirm this before the operation proceeds.
5. marking of the first secondary replica as valid in the Object Dictionary and replicating this change on all accessible nodes: The majority of the nodes must confirm this before the operation proceeds.
6. initiation of the creation of the rest of secondary replicas: These shall proceed asynchronously. No confirmations are awaited.
7. confirmation of object creation to the issuer of the request.

### 4.8. Changes in Object State and Their Replication

A change in the state of a DOORS object means the modification of at least one of its attributes. This may occur in one of the following ways:

- upon execution of one of its own methods: The body of the method may contain a statement in the form “set (attribute, new-value)”.
- upon receipt of a message requesting the modification of the respective attribute: DOORS only allows compare-and-set semantics; therefore, the message must be in the form “change (attribute, old-value, new-value)”. Such a request shall fail if the current attribute value does not equal the old-value indicated in the body of the message. Such a message may be received from either a client or from another object within the system.

A successful change of an attribute must be propagated to all object replicas, in semi-synchronous manner, as described in Figure 8. For maximum efficiency in the propagation of changes, the write message from the master node to the slaves is always in simplified form: “set (attribute, new-value)”. The primary value always takes precedence; therefore, we can do away with the compare-and-set semantics.

### 4.9. Failure Detection

Due to the chosen communication solution (based on TCP sockets and the event-based portable library *libevent* [22]), DOORS is able to directly detect only one type of failure: loss of communication. We managed to time-bound this detection, by implementing messages of known length, and discarding messages not received integrally in a set window of time. Details on the communication framework and implemented state machine can be found in Reference [5].

Whenever a message is sent, each of the two involved nodes get a fresh view of the status of the the other. The receiver node learns that the sender is accessible upon the receipt of the message, while the sender learns about the status of the receiver based on whether it receives a confirmation or not.

During periods of “silence”, the peer status information would “go stale”. To avoid this, in absence of useful traffic, DOORS nodes exchange status query messages regularly, such that any inaccessible node is detected with a known maximum delay. The fixed time period at which the status inquiry messages are exchanged is, therefore, the longest time interval for which the nodes are not aware of the status of their peers. This is completely under the control of the system; it does not depend on the periodicity of the useful traffic and is configurable in DOORS. Connection loss may mean anything, from transient communication issues, to software malfunctions, or even total loss of node hardware.

### 4.10. Reaction to Failure

When a node detects failure of at least one peer, it shall perform the following operations, as soon as possible:

- determine whether itself forms a majority with the all the peers it can still access: this status information is important for determining the behavior of the node during the recovery;
- establish the impacted set: this set contains all objects in the system for which their master replica is currently inaccessible from the current node.

Nodes which are not part of a majority (i.e., which can still access less than half of their peers or no peers at all) shall not perform election of a new master for the impacted set. Instead, all messages originating from their directly connected clients, as well as from the objects for which they are the master replica, and aimed at objects from the impacted set, shall be queued and kept locally. Any messages received (and which of course are aimed at objects for which the node is master) shall be processed normally.

Nodes which are part of a majority (i.e., which can still access at least half of their peers) shall perform the election of a new master, by iterating through the Object Dictionary entries pertaining to objects from the impacted set and automatically promoting to master status the first ID in the list of slave replicas, which is still accessible. In the example given in Figure 9, if node 5 becomes inaccessible, and the current node is part of the majority; then the new master node for the depicted object shall be node 3, and iff node 3 is still accessible from the current node. Otherwise, the new master node shall be node 8. If none of the indicated secondary replicas are in fact accessible from the current node, then, the respective object shall be marked as *inactive* and all messages aimed at them shall be locally queued.

The most probable type of failure is single-node isolation. This occurs when only one node loses its network connection and, therefore, becomes isolated from the rest of its peers. We shall analyze the effect of this failure upon replication, with the help of Figure 8:

- If N2 fails, then, N3 would be promoted it is place as soon as this condition is detected, and this is due to the fact that, according to Figure 9, N3 follows N2 in the list of secondary replicas. The update operation during which the N2 failure occurred would be then performed normally on N3 and the new slave replica that would have to be recruited. The state of N2 would be re-synchronized upon its “return to the system”, according to the outline in Section 4.10.2.
- If N3 fails, then, a new node would have to be recruited as slave replica and synchronized as soon as this condition is detected. The update operation during which the N3 failure occurred would be then performed normally on N2 and the new slave replica. The state of N3 would be re-synchronized upon its “return to the system”.
- The most complex case is when N1, the master replica, fails during the update of an object. In this case, the secondary replicas learn about the master failure due to lack of traffic (no response from N1 to normal requests sent by N2 or N3 or no normal requests coming from N1 or, in the absence of useful traffic, no status inquiries coming from N1). The changes required by the client and performed on N1 would not be persisted into the system only if N1 failed before sending the “write msg1”. In this case, the client would not have received the confirmation, and N2 would be promoted to master, while containing the old, not updated value. If, however, the N1 fails after sending “write msg1”, then, the N2 would be promoted to master with the updated value. The update would not be lost, but the client would not have received its confirmation. There would be a workaround, by issuing “write msg1”, “write msg2”, etc., after the completion of “local write 1”. However, this would have dramatic effect on the performance of the writes, and we chose not to implement this variant in DOORS.

#### 4.10.1. Recruitment of New Followers

At this moment, all objects from the impacted set will be temporarily having one less slave replica. The procedure of election of a new slave replica is in the charge of the newly elected master and shall be accomplished by random selection. The result of the selection shall be broadcast by the new master to the rest of the majority.

#### 4.10.2. Synchronization after Connection Restore

When any of the previously inaccessible nodes become accessible, a synchronization of Object Dictionaries shall occur. This phase relies on a dialogue between the two nodes and the details depend on previous state of each of the two, as follows:

- If one of the nodes was part of the majority, his Object Dictionary entries will take precedence over the entries from the Object Dictionary of the other node (which was for sure not part of the majority during the communication failure). After synchronization, both nodes are considered part of the majority [23].
- If none of the nodes was part of the majority, their synchronization shall be postponed until one of them becomes part of the majority. In the special corner case in which a majority is not achievable (due to the fact that the system was heavily segmented by the failure), a special session for construction of majority shall be initiated. The construction of majority, as well as the case of cascading failures, are not in the scope of this material.

After the successful synchronization of the Object Dictionaries, the nodes that joined the majority shall proceed to transmit, in order, all the messages queued locally.

As described in Section 4.10, DOORS nodes maintain the convention to promote to master the first of the secondary replicas. This implicit form of consensus is ensured iff all nodes have a consistent object dictionary. The complete list of operations which perform changes upon the Object Dictionary and, therefore, need a consensus operation in place are:

- creation and deletion of objects;
- migration of objects—please note that only the master replica of an object shall be moved;
- graceful node life-cycle events, e.g., an admin issuing a command for a node to leave or join a certain system instance; and
- communication failures, as described above.

### 4.11. Partitioning in Conjunction with Replication

Partitioning may and shall be used in conjunction with replication [24]. This way, the system gains both resilience through redundancy and capacity through horizontal scaling. This, however, complicates the system implementation, as commonly shared data structures must keep track of all replicas and their distribution across the system.

**Figure 10:**
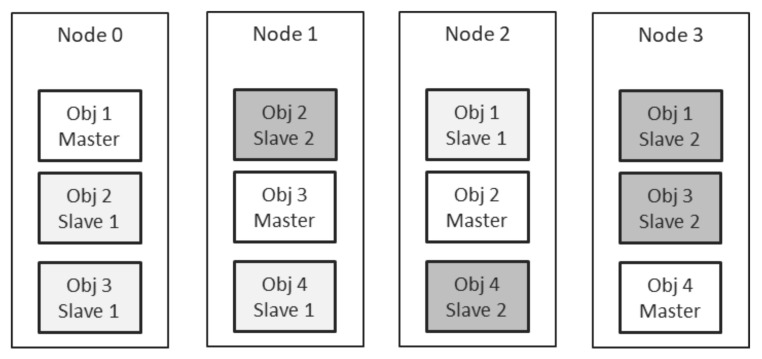
Partitioning and Replication in a DOORS instance.

Figure 10 shows an example DOORS instance, containing 4 nodes and employing a replication factor of 3. The system hosts 4 objects, and each of them has 1 master replica and 2 slave replicas. The replication mechanism ensured uniform distribution of replicas, while the balancing algorithm ensured uniform distribution of the master replicas of each object.

## 5. Experiments

### 5.1. The Test Setup

In the context of the *Minimal System* [4], the first operation performed by a generic DOORS application is creation of objects. This involves the actual allocation and initialization of the object, as well as the correct initialization of the Object Dictionary [25].

Our first experiments test the capacity and scaling capabilities of the set of data structures chosen to store the Object Dictionary. The test platform runs Manjaro Linux 21.1.2, with kernel version 5.10 on a dual-socket machine, with a total of 24 threads running at 3.06 GHz and 32 GB of RAM.

### 5.2. Initialization of the Object Dictionary

**Figure 11:**
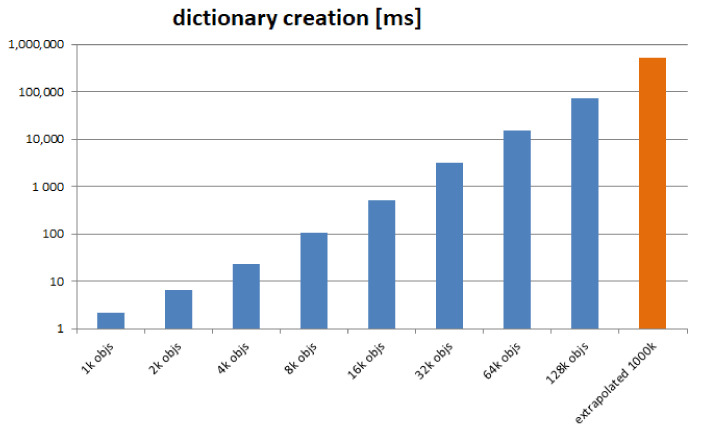
Object Dictionary creation time, logarithmic scale.

We performed 8 rounds, with increasing number of objects. We start with 1000 objects (all identical, and instances of the same minimal class, as described above) and continue in geometric progression up to 128,000 objects. We measure the time required to complete the set-up. The results are depicted in Figure 11.

The final value is extrapolated, based on the recorded series. We see a duration of 72 s for creating a dictionary of 128,000 objects and extrapolate to almost 9 min for creating a dictionary of one million objects. The conclusion is that our currently adopted solution, of creating the dictionary “from scratch”, does not scale satisfactorily. DOORS must build the dictionary incrementally and rely on storage in order to become a usable system.

### 5.3. Status Inquiry

**Figure 12:**
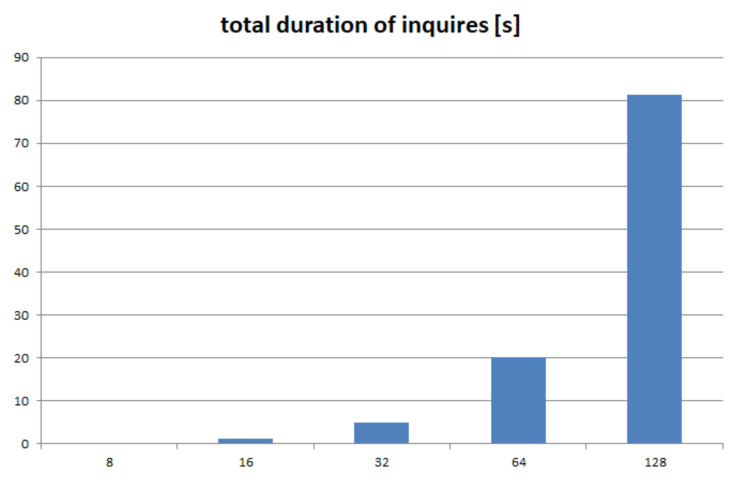
Evolution of the total duration of status requests as a function of the number of peers in the LS.

As described in Reference [3] and based on the solution outlined in Reference [26], each DOORS node exchanges “heartbeat” messages with all its peers in order to detect communication failures. The drawback of this approach is of course the low scalability (the cost increases linearly with the number of peers). According to measurements performed in a non-perturbed 780 Mbps Wi-Fi network, the duration of one status inquiry is around 10 ms and involves the transmission of less than 60 bytes (it involves three one-way trips between the nodes). Given the fact that one message exchange yields status for both involved nodes, the number of messages needed for a full status update of an *N*-node system is N·(N−1)/2. The evolution of the total duration of the status update as a function of the number of peers is depicted in Figure 12.

A DOORS Local System of 128 peers would then need 90 s just to get a full status update. One must consider then the occupancy of the communication channels. If we would restrict the status inquiry traffic to a maximum of 25% of traffic, it would mean that we would never be able to get a status update more often than any 325 s. Fortunately, a solution to this limitation is immediate: the status inquiries shall only be exchanged in the absence of useful traffic.

By periodically computing latencies and by exchanging status information, the nodes shall be able to distinguish between:

- system segmented (each node is alive, but part of one of potentially several segments);
- node isolated (a single node is missing, or the present node is active but isolated); and
- perturbed network (multiple transient node disappearances).

### 5.4. The Cost of Election of a New Master

**Figure 13:**
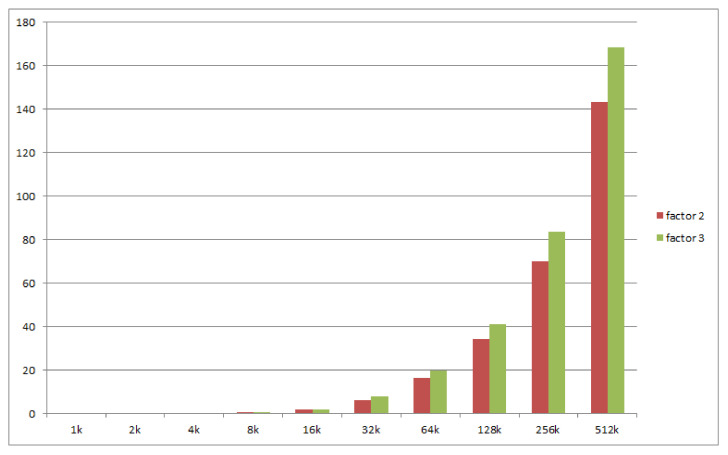
Election of new master for unavailable objects.

We measured the cost of replication and its evolution as a function of the replication factor and of the number of objects. We use a geometric progression in the number of objects: 1000, 2000, 4000, all the way up to 512,000 objects. We perform 2 sets of measurements for replication factors of 2 and 3. The results are depicted in Figure 13.

The cost of master election for 512,000 objects with a replication factor of 3 is less than 170 ms. By extrapolating the series, we find out that, for a cost of 1s, the system is able to manage the replication of around 3.6 million objects. For future practical cases, in which this cost is not acceptable, we shall consider optimization of the election algorithm.

The actual values recorded are listed in Table 1. The values show a relatively linear growth. Doubling the number of objects yields a roughly twice as long election time. This is consistent with our estimation, based on the type of data structure currently implementing the Object Dictionary, which is a double-linked queue, for which the traversal is O(n). We have, however, exceptions, for numbers of objects equal to 16 k up to 64 k, for which the duration increases more rapidly. We consider that the cause lies in the “loss of locality”. These are the sizes at which the data structure no longer fits in the CPU caches and performance degrades. For numbers greater than 64 k objects, the recorded duration grows linearly again.

## 6. Conclusions

The paper presented the replication solution for the object-oriented runtime system for Edge Computing. The solution is semi-synchronous with a configurable factor having the capability to move objects from one node to another. We described the structures of objects and classes in DOORS, the definitions for attributes, methods, and parameters, and the communication protocols. The communication issues are also presented, and proper solutions are adopted. The partitioning aspect is analyzed in conjunction with DOORS’ nodes replication. The experimental results show the ability to distinguish between system segmented, node isolated, and perturbed network.

A series of baseline experiments were performed and documented, measuring the duration for: the creation of the Object Dictionary, the system-wide status inquiry, and the cost of replication. Besides establishing a quantitative baseline, the experiments conclude that:

- “full-rebuilds” of the Object Dictionary should be avoided; instead, this system data structure shall be saved into persistent storage and restored from there upon node restart;
- status inquiry grows rapidly with the number of nodes; in order to scale acceptably above 100 peers, nodes should only use dedicated “heartbeat” messages only in absence of timely useful traffic; and
- the relatively naive implementation of the Object Dictionary in the DOORS Minimal System is capable of electing new master replicas for 3.6 million objects per second.

As future work, we may focus on migration of client connection, efficient dictionaries, polymorphism, and event-oriented language implementation. 

## Figures and Tables

**Table 1:** Duration measured for the election of new masters for unavailable objects.

Number of Objects	1 k	2 k	4 k	8 k	16 k	32 k	64 k	128 k	256 k	512 k
**Election time, r = 2 [ms]**	0.081	0.167	0.3	0.7	1.844	6.395	16.365	34.358	70.148	143.477
**Election time, r = 3 [ms]**	0.092	0.188	0.378	0.795	2.031	7.734	19.703	40.955	83.676	168.486

## Data Availability

Not applicable.

## Abbreviations

The following abbreviations are used in this manuscript:

AAL	Ambient Assisted Living
DOORS	Distributed Object Oriented Runtime System
GUID	Globally Unique Identifier
IoT	Internet of Things
OOP	Object Oriented Programming
SCADA	Supervisory Control and Data Acquisition
